# Socio-spatial relations observed in the global city network of firms

**DOI:** 10.1371/journal.pone.0255461

**Published:** 2021-08-17

**Authors:** Thomas Sigler, Kirsten Martinus, Julia Loginova

**Affiliations:** 1 School of Earth and Environmental Sciences, University of Queensland, St Lucia (Brisbane), Queensland, Australia; 2 School of Social Sciences, University of Western Australia, Crawley (Perth), Western Australia, Australia; University of Lausanne, SWITZERLAND

## Abstract

One of the prevailing approaches to the study of the global economy is the analysis of global city networks based on the activities of multinational firms. Research in this vein generally conceptualises cities as nodes, and the intra-firm relations between them as ties, forming the building blocks for globally scaled interurban networks. While such an approach has provided a valuable heuristic for understanding how cities are globally connected, and how the global economy can be conceived of as a network of cities, there is a lack of understanding as to how and why cities are connected, and which factors contribute to the existence of ties between cities. Here, we explain how five distinct socio-spatial dimensions contribute to global city network structure through their diverse effects on interurban dyads. Based on data from 13,583 multinational firms with 163,821 international subsidiary locations drawn from 208 global securities exchanges, we hypothesise how regional, linguistic, industrial, developmental, and command & control relations may contribute to network structure. We then test these by applying an exponential random graph model (ERGM) to explain how each dimension may contribute to cities’ embeddedness within the overall network. Though all are shown to shape interurban relations to some extent, we find that two cities sharing a common industrial base are more likely to be connected. The ERGM also reveals a strong core-periphery structure in that cities in middle- and low-income countries are more reliant on connectivity than those in high-income countries. Our findings indicate that, despite claims seeking to de-emphasise the top-heavy organisational structure of the global urban economic network, interurban relations are characterised by uneven global development in which socio-spatial embeddedness manifests through a combination of similarity (homophily) and difference (heterophily) as determined by heterogeneous power relationships underlying global systems of production, exchange and consumption.

## Introduction

The inter-urban relations of firms are a commonly used proxy indicator for understanding the global economy from a network perspective. Though several network-based approaches to the study of the global economy exist [1–3], the ‘global city networks’ approach is rather well-established, with several interrelated methodologies seeking to explain global urban hierarchies, sub-networks, and the relationships between world cities and regions [4, 5]. Its theoretical and methodological attempts to understand how cities are globally networked [6] have been deployed by spatial scientists from various disciplinary backgrounds, and increasingly by network theorists, whose sophisticated analysis addresses systemic complexity in progressively nuanced ways [7–9].

The global city networks literature has primarily focussed on the application of social network analysis (SNA) to uncover the relational nature of transnational urban connectivity [10, 11]. In general, it focusses on the multi-locational geographic strategies of multinational corporations (MNCs) [12] as a proxy for a number of broader economic processes tied to globalisation. Coinciding with the popularisation of global city networks research in the late 1990s, the globalisation of MNCs has been a major driver of this research, both as a topic of interest as well as a rich source of data. MNCs are, by most accounts, the fundamental building blocks upon which the global economy is organised [13, 14], directing to a large extent the international division of labour, and exerting more financial power than the sum of global governments. The operations of many contemporary MNCs span across multiple cities, countries, and indeed continents, and the invisible complex network of headquarters, branch offices and subsidiaries is produced by flows of information, knowledge, capital, and other resources [15, 16]. Given the systemic properties of such office ‘networks’, the application of complex network theory [17–19] presents attractive analytical methods for investigating the global city network through the lens of MNCs.

A major recognised limitation of existing research on global city networks of firms is an inability to account for multiple and alternative globalisations that unfold across the world [20–22]. Researchers have pointed out that there is no singular global city network of firms, but rather multiple overlapping network layers, each of which is shaped by its own irregularities and relationships [12, 20]. As both Derudder [23] and Neal [24] contend, there is a risk that certain approaches reify *the* global city network as an actually-existing and singular construction rather than as an heuristic for understanding networks’ underlying processes. To this end, Derudder [23] suggests that geographers should no longer borrow from the social network analysis literature, but instead attempt to explain global city networks in terms of how socio-spatial processes shape the distribution of MNCs and vice-versa. Moreover, while network theory and science has continued to evolve, the global city networks literature has somehow failed to incorporate a multitude of sophisticated network methodologies to explain underlying economic processes. While there are of course exceptions [12, 20], the processes underlying global city networks have to date largely been explained by qualitative and/or descriptive approaches (e.g. global production networks, or GPNs), or by an artificial bifurcation of cities into those at the upper echelons of city networks (e.g. ‘world cities’) and ‘ordinary’ cities, whose underlying economic functions are more quotidian or less global [25].

This paper addresses a theoretical and empirical gap in how we understand global city networks by attempting to explain how underlying socio-spatial processes influence network structure and composition. We draw upon five distinct node-level dimensions to help explain the absence or presence of dyadic relations between cities as a function of firms’ embeddedness within global systems. The paper first sets forth our hypotheses relating to each of these five dimensions, focusing on how individual cities’ relations are a product of each. In contrast to multiplex analysis, which stitches together multiple network layers, we begin with a single global city network layer constructed from a two-mode network of firm-subsidiary ties. After demonstrating how the connectivity of individual cities within the overall network is comprised of distinct combinations of these five dimensions, we then apply an exponential random graph model (ERGM) to explain dyadic relations between cities. Our analysis attempts to conceptualise the multirelational nature of the global city network of firms along the dimensions of regional, linguistic, industrial, developmental, and command & control relations, operationalised by node-level variables explaining dyadic interurban relations. Our approach attempts to bolster the literature on how multiple globalisations explain global city networks of firms by focussing on the dimensions that explain inter-urban economic connectivity.

### Global city networks of firms

Research on global city networks of firms is rooted primarily in the structuralist tradition of international political economy. Several inter-related disciplines have emerged in this tradition, including international business [14] with a more firm- and institution-centric focus; economic globalisation literature [26] centred on geopolitics and trade; and economic geography [27], with its emphasis on spatial processes and outcomes. Within economic geography, efforts to theorise the spatial character of the global economy have fallen into one of several primary approaches, including global commodity chains [28], global value chains [2], global production networks [29, 30], and global city networks, often referred to as ‘world city networks’ [10, 31]. Of these, the global city networks approach has the most quantitatively grounded empirical focus. Though in fact capturing a range of interrelated methodological and theoretical approaches, research in global city networks generally comprises a common core focus on the international activities of MNCs [12, 32]. The initial focus of this approach was on advanced producer services firms (e.g. law, consultancy, finance), yet more recently the interlocking world city network model (IWCNM) [33, 34] has been applied to new sectors [21, 27], and modified to accommodate new data sets [35], new methods [36], new theoretical questions [37], and to explore beyond the upper echelon of so-called global cities [38, 39].

The global city networks approach is simultaneously a methodology, a theory, a hierarchy, and a novel nomenclature [40]. Notwithstanding, perhaps the most well-known outcome of research on the global city networks of firms has been a better understanding of the transitive and relational economic processes that shape global cities, and how this changes over time. Beyond the mere observation that New York, London, Paris, Hong Kong, and Tokyo sit atop hierarchical league tables, network metrics have revealed several nuanced details including the respective ‘command & control’ regional aspects of these cities’ economic hinterlands [31], industries that are formative of particular relationships [21], and places in which economic connectivity is disproportionate what may be anticipated based on an industrial base or population size alone.

Aside from the global city networks literatures, a number of parallel approaches concentrate specifically on place-based networks derived from economic data, with foci on input-output, trade flows, passengers flows and other ‘real’ relational data [41, 42]. Unlike these, the global city networks approach takes a more heuristic perspective on networks in that relations are comprised of the relationships between headquarters offices and their branches or subsidiary locations. Thus of the numerous critiques waged against global city networks research, one of the most pronounced is a lack of clarity around the nature of ‘ties’ as bilateral relations between cities [43].

Various readings have alluded to conceptual weakness in that the “interlocking approach can structurally predetermine features of the resulting world city network” [44] (p. 63). According to Nordlund [45], part of the problem lies in obtaining appropriate data, yet this “cannot be alleviated by generating artificial data sets based on internal attributes of the actors” (p. 295). Despite this deficiency, however, most research on global city networks of firms continues to “rely on a legacy of using data on office locations of firms to indirectly estimate intercity business flows” [46] (p. 1). Such methodologies assume that information, capital, and other resources are transmitted from one location to another, and but there are no assumptions of hierarchy between cities containing global firm headquarters. Though this may be the case, interpretations are parsimonious in their ability to disentangle the various socio-spatial that shape ‘global’ networks, and to explain multiple globalisations vis-a-vis networks.

### Approaches to understanding ‘multiple globalisations’ in global city networks of firms

To address the paucity of literature demonstrating how global city networks of firms are shaped by multiple globalisations, there are several distinct approaches. The first is a comparative approach to understanding multiple networks, based on a common economic dimension. This may entail comparing city networks constructed from multiple industry sectors [39], or a single industry sector [21] across time or space [47]. Such an approach can often be quite descriptive [20], using network terminology more as an heuristic than in a formal manner. Moreover, this approach focusses more on nodes (cities) and network structures rather than on ties.

A second approach is captured by the diverse literatures applying the concept of multiplexity [48, 49]. In multiplex networks, each ‘layer’ comprises a distinct network, whose sum amounts to the ‘global’ network composed of multiplex relations. Verbrugge [50] defines multiplexity within social networks as ‘the co-occurrence of distinct roles’ or the ‘multiple bases for interaction’ in a dyad (or ties between a) pair of nodes or actors (p. 1287). Therefore, in a conventional ‘social’ network, it is possible to simultaneously be a neighbour, a friend, a relative, and a co-worker of another individual, meaning that a tie is concomitantly linked to multiple phenomena. Though initially relating to interpersonal networks, multiplexity has more recently been applied to explain economic relationships. For example, Ferriani et al. [51] found that although both social and economic ties increase the likelihood of tie formation between firms, social links tend to be more significant than economic ones. However, we eschew this approach because of the difficulties in identifying, measuring, and interpreting multiplexity within large-scale global networks [52].

In this paper, we apply a third approach, which is the explanation of how node-level attributes help explain the socio-spatial processes underlying dyadic relations between cities in global networks. We specifically focus on the determinants of ties in order to address what we perceive to be a significant lacuna in the literature, namely a lack of quantitative analysis to explain interurban relations. Using an exponential random graph model (ERGM) to analyse homophily and heterophily in dyadic relations between nodes, this approach is applied to better understand whether cities tend to connect depending on their attributes. This is a common approach in SNA, previously applied to the study of social phenomena such as farmers’ trade networks [53] or policy networks [54]. More detail on this technique is provided below in the methodology section.

### The embeddedness of global city networks of firms

As our analysis is primarily concerned with better understanding the socio-spatial dimensions that explain dyadic relations in global city networks of firms, we theorise network relations to be ‘embedded’ in a number of broader, complex systems. Embeddedness refers to the concept that economic behaviour is ‘socialised’ by non-market relations. In economic sociology (from which economic geography is in part derived), network embeddedness occurs via processes that bring two nodes together to form a tie that persists across time and space [55]. Initially observed as part of the substantivist position on the relationships between social and economic relations [56], embeddedness provides theoretical explanation for firm strategy and behaviour that links cities in global networks. Firms do not select cities in which to establish subsidiaries at random, and therefore the shape and structure of the overarching global city network of firms reflects the sum of these non-random decisions that are constrained by institutional, political, economic, and other factors. In this instance, we operationalise these as socio-spatial dimensions, explained in the following section.

Rivera et al. [55] argue that there are three key change mechanisms facilitating embeddedness in network analysis: 1) assortative, which depend on actor attributes (compatibilities, complementarities); 2) relational, which influence the network position and relations of an actor (e.g., gained through trust, introductions, knowledge); and, 3) proximity, which is gained through the social organisation of interactions. These have been frequently measured through social network analysis (SNA), whereby homophily or heterophily identify proximity and assortativity; reciprocity, repetition, clustering or degree identify relational mechanisms (e.g., [57]).

Our analysis takes these mechanisms into account, by way of five dimensions operationalised by node-level attributes. In other words, firms’ economic behaviours (i.e. whether to locate a subsidiary in another city) are conditioned and influenced by a multitude of factors. Each of these is described in the following section, along with five hypotheses [H1, H2, H3, H4, H5] relating to the effect each may have on dyadic relations between two cities.

### Dimensions of global city networks of firms

The structure of global city networks of firms is determined by the interconnectedness of cities. Just as in all networks, there are certain observable properties in the global city network of firms that we describe below, notably the tendency for a small number of ‘world cities’ to be highly connected (preferential attachment). Dyadic connectivity between cities is reliant on the existence of firm relations, which in this case we operationalise as firm-subsidiary relations. If there is a firm location in one city and its subsidiary in another, the two cities are connected.

We draw upon the following dimensions to explain socio-spatial embeddedness within global city networks of firms: regional, linguistic, industrial, developmental, and command & control. Each dimension is conceptualised as a node-level attribute and is supported by literature explaining how and why we may anticipate a tie based on either homophily or heterogeneity.

### Homophilous socio-spatial dimensions

With regard to the **Regional Dimension**, we hypothesise that in the global city networks of firms cities tend to form more network ties with cities in the same world region [H1].

This homophilous relationship is based on certain regional commonalities or interests. The regional dimension is perhaps the easiest to understand because it hinges on geographical proximity. As Tobler’s first law of geography states, “everything is related to everything else, but near things are more related than distant things” [58]. Spatial networks tend to exhibit higher clustering coefficients than compared to non-spatial networks due to the high importance of proximity in node connectivity [59].

The regional dimension binds formal and informal geographic ‘regions’ together through connections between people, places, and institutions within a city, province, country, or part of the world. In this instance, we apply the World Bank Regions classification [60] (East Asia & Pacific, Europe & Central Asia, Latin America & Caribbean, Middle East & North Africa, North America, South Asia, and Sub-Saharan Africa) as the working definition of regional boundaries.

Spatial relations are reinforced by trade agreements and trade blocs (e.g. NAFTA, ASEAN, MERCOSUR) which connect neighbouring countries through tariff reductions, migratory accords, and so on [61, 62]. Rozenblat et al. [12] find that regional relationships are reinforced by multipolarity, which increases along with firms’ level of technological sophistication.

With regard to the **Linguistic Dimension**, we hypothesise that in the global city networks of firms cities tend to form more network ties when they are located in countries sharing a common language [H2].

This is a homophilous assumption [57], with common language assumed to facilitate business transactions. Evidence of this is buttressed by the manifold impacts of a common language applied to digital communication to overcome distance, for example communication by email or social media [63, 64].

Common language is generally derived from one of two historical artefacts, both of which explain firms’ social embeddedness to some degree. The first is territorial expansionism indicating a common language spoken by geographical neighbours, as is the case from the Maghreb to the Arabian Peninsula (Arabic), from Eastern Europe to the Pacific Ocean (Russian), and spanning the centre of continental Europe (German). The second relates to colonial expansion, linking both near and distant lands through linguistic ties often relating to former or present relations, which Shaw [65] argues can be significant in linking nations not only through language, but through common cultural practices (e.g. sport), governance structures, infrastructures (e.g. transport networks), and social media. This is articulated in various ways, such as diasporic linkages for both permanent and temporary (e.g. tertiary education) migration, and more subtle ones such as rugby and cricket rivalries between distant pairs, for example West Indies and New Zealand. Though English has rapidly become the world’s *lingua franca* of business [66], other linguistic connections foster circuits of migration, education, and firm activity, for example Francophone West Africa to France, and Lusophone connections between Angola, Brazil, and Portugal [67]. Linguistic ties as socio-cultural linkages are reflected in the co-residential choices of migrant groups [68], whose diasporic linkages often foster and support business networks to the ‘mother country’. In this case, we use linguistic classifications according to the CIA World Factbook [69] and select the first major language of a country.

With regard to the **Industrial Dimension**, we hypothesise that in global city networks of firms, cities tend to form more network ties when they share a dominant industry [H3].

This is a homophilous assumption [57] in that cities are often bound together by firms whose activities are functionally complementary. For example, there may be higher-than-average connectivity between ‘college towns’, whose students and academics visit with one another, or between cities involved in the global automobile industry, as components suppliers require relationships with assembly plants, and vice versa. Voluminous research has brought to light these connectivities, producing distant pairwise relations between cities involved in oil and gas [70], finance [71], information technologies [72], and so on. Sister city relations are another tangible example of functional network connections [73, 74]. For example, Pittsburgh enjoys sister city relations with other steel producers such as Saarbrücken, Sheffield, and Bilbao, while Los Angeles is paired with others whose presence is notable in the film industry, such as Vancouver and Mumbai.

The industrial dimension perhaps the least spatially articulated, and can be conceptualised as one that draws places together through processes of strategic coupling, or common economic or intellectual benefit. According to the theories of self-interest, actors form and uniform links based on strategic decision making and evaluation of the cost and benefits of international interactions [75].

The industrial dimension has geo-political aspects given the importance of resources and trade to nation-state interests. For example, Martinus and Tonts [70] have found that Australian corporate energy networks largely conform to national and regional energy systems, but that ties often connect functional systems across great distances. Ng and Soo [76] identify alliances using weapons trade data, suggesting that industry-based connections between countries involved in the global arms trade are not freely formed, but rather strongly shaped by geo-political factors resulting from mutual benefit. To analyse the functional network, we used network relations between cities with a majority of ties within the same industry, according to the Statistical Classification of Economic Activities in the European Community, known as NACE (Nomenclature des Activités Économiques dans la Communauté Européenne).

### Heterogeneous socio-spatial dimensions

In the search for new markets, both in terms of inputs as well as markets and consumers, global city networks reflect firms’ abilities to leverage and exploit wage differentials and other economic disparities, such as resource endowments, through uneven and hierarchical relationships. Wallerstein’s [77] World Systems Theory characterises the relationship by which less-developed (periphery) regions are reliant on the core and semi-periphery for technologies and capital, and more developed (core) regions are reliant on the semi-periphery and periphery for labour and raw input materials. There are therefore two equally significant components of ‘unevenness’ related to the final two hypotheses: development (proxied by income inequalities) and command & control (proxied by city centrality by MCNs). As many have observed [78] cities–conceptualised as ‘natural’ systems–have a tendency to be organised hierarchically along several dimensions.

With regard to the **Development Dimension**, we hypothesise that in the global city networks of firms, cities tend to form more network ties when they are located in two countries with disparate levels of income than if they are located in two countries with the same level of income [H4]. This prioritises heterophily over homophily in that there is complementary between higher and lower income countries. Although Wallerstein’s contemporaries have voiced a number of qualms with these dependency structures, there remains a large disparity in income levels between countries and regions, contributing substantially to the new international division of labour [79] and global distribution of economic activity more broadly.

In differentiating this dimension from others, Guiliani and Pietrobelli [80] note that the embedded power structures of global hierarchies are more important in some networks than in others. From a network theory angle, Bonacich [81] suggests that in “bargaining situations, power comes from being connected to those who are powerless, as being connected to powerful others who have many potential trading partners reduces one’s bargaining power” (p. 1171). The development dimension thus derives its structure from global inequality whereby cities in low-income countries are more connected to cities in high-income countries than elsewhere in their region. To examine the development layer, we analysed the relationships between cities in different World Bank Income Groups (Low Income, Middle Income, and High Income) classification [60].

With regard to the **Command & Control Dimension**, we hypothesise that in the global city networks of firms cities tend to form more network ties when cities exhibit disparate levels of nodal importance than those with similar levels of nodal importance [H5]. This disassortative relationship is characterised by two cities with different levels of degree centralities (High and Low). This assumes intrinsic benefit to heterophily, in that firms in less-connected cities may benefit from connectivity to firms in more-connected cities, and vice-versa. By the same token, firms in ‘world cities’ benefit from connectivity to one another, given both cities’ relative urbanization and agglomeration economies.

Much of the literature on global city networks of firms has observed a distinct command & control [82] geography in which large MNCs agglomerate in a small number of ‘global cities’. New York, London, and Tokyo have been reified as such in the works of Sassen [83] and many others affiliated with the Globalization and World Cities (GaWC) research network. Though some research has sought to de-bunk the ‘myth’ of command & control [84], the global city networks observed across hundreds of published papers still exhibit a persistent top-to-bottom connectivity pattern, in particular favouring cities housing firms in advanced producer services (APS).

The theory behind the global city command & control network is explained by two interrelated network phenomena. The first is preferential attachment [85], which explains why nodes (cities) with high connectivity become increasingly more connected at the expense of less connected nodes (cities) in the network. In the event of a new market entrant (in this case, a new firm office or subsidiary) there is a disproportionate likelihood of the MNC choosing a global city as its geographical location, suggesting that locations in less-connected cities will statistically favour those in more-connected cities. The second is the concept of scale-free networks, meaning that as the network expands or contracts, its underlying structure remains the same. Again, this supports a ‘command & control’ network by assuming more-connected nodes (cities) will maintain their level of connectivity, and aside from macro-structural ‘shocks’ to the system, the network will retain highly uneven relations between well-connected nodes and the poorly connected (network) periphery.

## Data and methods

Corporate ownership data was obtained from the Osiris database made available online by Bureau van Dijk [86], in this instance access through The University of Queensland’s institutional license. The database offers detailed information on approximately 80,000 public companies listed on 208 global stock exchanges, including information about their subsidiaries. In particular, the database contains addresses of most firms and industry classification of their activities. At the start of the analysis, we obtained information on 66,263 headquarters and 965,995 subsidiaries, including their industry classification using the European industry classification standard NACE (Level 1).

Entries with missing addresses and NACE codes were excluded. We then batch-geocoded the location of each parent office and subsidiary using the Google Maps API. The geolocated addresses were assigned to metropolitan areas based on a ‘world urban areas’ file obtained from the UCLA Institute for Digital Research and Education [87] created by ESRI Data and Maps. Firm locations were reclassified as the closest large city in the same country if they fell within a buffer polygon of 75 kilometres for cities of 1+ million population, 50 kilometres for cities of 100,000+ population and 25 kilometres for smaller cities. Firm and subsidiary points falling outside of these polygons were not reclassified. This ensured that suburbs of large cities were reclassified to indicate that they were part of a larger metropolitan area. The large number of global subsidiaries outside of major cities (e.g. production plants or mine sites in rural areas) explains why the list of cities in our cities matrix was significantly larger than in previous similar research.

Once reclassified, interurban ties were identified if two cities had a headquarters-subsidiary ownership relation. In total, 822,383 ties were identified, connecting 4,702 cities. To build the global city network of firms from these data, we included only subsidiaries with more than 50% of total ownership. Further, we included only firm relations between cities (by omitting intra-city relations) and we excluded domestic ties (when headquarters and subsidiaries are located in the same country) to account only for international connectivity. A weight was assigned to each inter-city tie based on the count of headquarters-subsidiary relations. As a result, 38,877 weighted city ties were constructed from corporate relations between 163,821 subsidiaries reporting to 13,583 headquarters. These directed ties span across 3,042 cities in 187 countries and territories (as defined by unique ISO non-sovereign country codes). The resulting global city network of firms is a one mode directed network graph *G* = (*N; E*) with *N* = 3,042 nodes (cities) and *E* = 38,877 weighted ties.

Each node (city) was assigned five categorical attributes, according to the specification in Table 1. Three of these were enumerated at the country level (world region, primary language and income group) and two at the city level (international industry specialisation and measure of city’s command & control based on its degree centrality in the network).

**Table 1:** Socio-spatial node-based attributes in the global city network of firms.

Attribute	Data (Spatial Scale)	Data description	Effect	Type of socio-spatial relation	Example
World region	World bank regions classification [60] (Country)	East Asia & Pacific Europe & Central Asia Latin America & Caribbean Middle East & North Africa North America South Asia Sub-Saharan Africa	**Homophily**	**Regional**	Sydney-Beijing tie is homophilous (both cities are in the East Asia & Pacific region), Chicago-London is not
Primary language	World Factbook’s language classifications, including 78 languages coded using ISO 639–1 [69] (Country)	The primary language (official or major) spoken in a country	**Homophily**	**Linguistic**	Sydney-London tie is homophilous (English is a common language), Paris-Beijing is not
International industry specialisation	NACE codes as listed in the Osiris database [86]: Any city with at least 5 international ties and an industry share greater than 50%, as defined by the count of headquarters and subsidiaries in this city. (City)	14 NACE industry groups (Level 1)	**Homophily**	**Industrial**	Linz-Chicago tie is homophilous (both cities specialise in Manufacturing)
Income group	World Bank Income Groups classification [60] (Country)	High income Middle Income Low Income	**Heterogeneity** *Homophily* (similar level of development) *Heterophily* (different levels of development)	**Development**	Sydney-London tie is homophilous (High-High), Sydney-Jakarta tie is heterophilous (High-Low)
Degree	Ranking of cities based on the degree centrality (City)	High (top 10% cities with the highest values of degree centrality) Low (the remainder of cities)	**Heterogeneity** *Homophily* (similar levels of connectivity) *Heterophily* (different levels of connectivity)	**Command-and-control**	London-Saskatoon is heterophilous (High–Low), Paris-London (High-High) is homophilous

We performed ERGMs to test the effects of homophily (connectivity *within* attribute) and heterogeneity (connectivity *within* and *between* attribute) on the formation of the global city network of firms. As shown in Table 1, the effects of homophily are expected in ‘regional’ (each node in a dyad belongs to the same region), ‘linguistic’ (nodes in a dyad share a common language) and ‘industrial’ (nodes in a dyad have the same international industry specialisation) types of socio-spatial relations. The effects of heterogeneity are expected in ‘development’ (between cities characterised by the same *and* different income groups) and ‘command-and-control’ (between cities characterised by the same *and* different levels of connectivity) types of socio-spatial relations.

### Method

ERGMs are statistical models that test whether an observed network shows theoretically hypothesised structural tendencies, and are commonly applied in SNA [88, 89]. An ERGM can concurrently model network structure and the effects of nodal attributes on network formation. In many ways, ERGMs are analogous to regression, in that they explain independent variables against a dependent variable, which in this case is the presence of a dyadic relation between cities. In particular, ERGMs allow to incorporate categorical nodal attributes [90] and can be presented in the following form:

$$\mathrm{Pr}\left(X=x\right)=\frac{1}{k}exp(\sum_{A}\theta_{A}Z_{A}(x,Y)$$

where *X* = [*X*_*ij*_] is a 0–1 matrix of random variables representing network ties, *x* is a realization of *X*, *A* is a configuration, a (small) set of nodes and a subset of ties between them, *z*_*A*_(*x*) is the network statistic for configuration *A*, *θ*_*A*_ is a model parameter corresponding to configuration *A*, *Y* is a vector of nodal attributes, *κ* is a normalizing constant to ensure a proper probabilistic distribution. If a *θ*_*A*_ parameter is estimated to be significant, this suggests that the corresponding configuration has a greater chance of occurrence in the network, suggesting that the corresponding effect plays an important role in the network structure. This analytical technique enables us to test if intercity ties take place randomly or if nodal attributes (such as region) determine which cities connect.

Fitting ERGMs has become a common analytical strategy for modelling social networks. However, there are certain conceptual and computational issues with fitting ERGMS on large complex and real networks (more than a few hundred nodes) [91, 92]. Estimating parameters for large networks is a computationally difficult and time consuming, particularly for directed networks. Several solutions to these problems have been formalised, focusing on statistically complex modelling of a complete network or using a sample-based estimation of parameters followed by a meta-analysis, such as taking snowball samples from the original network [92, 93]. Structural reduction of a network is a common strategy to reduce the complexity and facilitate analysis. Indeed, when analysing a large graph with a skewed degree distribution and a low density, we may wish to focus on the core structure of the network. Therefore, we considered two common ways to reduce the network and extract its backbone. First, we removed all pendants, or nodes connected to the graph by only a single (in- or out-) tie. Sometimes ERGMs will improve and will fit when just the isolates and pendants are removed [94]. The decision to reduce the network has an effect on the descriptive statistics and subsequently on ERGMs (see Table 2). Removing pendants leads to a decrease in possible dyad count (from 9,238,560 to 4,278,692) and an increase in network density (from 0.004 to 0.009). Network density corresponds to the ratio between the number of city ties in the network and the number of all possible ties, when all cities were to be connected. Although achieving higher average degree and network density, such thresholding systematically discounts low-degree vertices therefore arguably alters important features of the network [95].

**Table 2:** Descriptive statistics illustrating the effects of structural reduction.

	Original network	Reduced network: No pendants	Backbone: Disparity filter (α = 0.05)	Backbone: Disparity filter (α = 0.05) Bonferroni corrected
**Edges**	38,877	18,966	3,197	267
**Nodes**	3,040	2,069	749	140
**Average degree**	25.577	36.646	8.537	3.814
**Density**	0.004	0.009	0.006	0.014
**Count of possible dyads**	9,238,560	4,278,692	560,252	19,460
**ERGM M0**				
**~Edges**	-5.467*** (0.005)	-4,717*** (0.005)	-5.161*** (0.018)	-4.275*** (0.061)
**AIC**	502,964	433,838	39,410	2,823

Note: Standard errors are reported in parenthesis and ρ-values are reported to the right of each coefficient.

**p < 0.05

***p < 0.01.

An alternative method is to apply filters that assign a ρ value to each edge using a null model of edge weight distribution and filtering out edges with the smallest ρ values (least likely to occur due to pure chance) [96]. The disparity filter is considered as one of the most effective backbone extraction methods [97]. This method statistically evaluates all edges of a given node in relation to one another. By imposing a significance level α, the edges that are considered not compatible with a random distribution are filtered out with the statistical significance. When applied to our original network, the disparity filter reduces the number of edges (and nodes) significantly, from 38,877 to 3,197 (with the significance level 0.05) (Table 2). Therefore, extraction of a backbone based on the disparity filter is less likely to underrepresent low-degree vertices and higher orders of tie dependence: network density and average degree are lower than in case with pendant-based backbone extraction method. Fitting a baseline ERGM M0 (with an *edge* parameter) in the network backbone yields a significantly smaller Akaike Information Criterion (AIC) (smaller is better) which is a measure of model fit based on variations on the deviance of a model. A negative *edge* term indicates that edges are not likely formed at random, as commonly found in real networks. Further, a Bonferroni test was conducted to modify the significance criterion (α/*k where k is the number of statistical tests*). The resulting Bonferroni network included 267 edges and 140 nodes, representing a statistically significant reduced network of the global city network of firms which was used to fit ERGMs as the models fitting less reduced networks failed to return an adequate goodness-of-fit. Since only a few cities (relative to the network size) represented Low income countries, they were combined with cities from Middle income countries in one group ‘Lower Income’ in the analysis.

After the baseline model M0, we fit an ERGM model M1 with parameters testing homophily and heterogeneity effects on the observed covariates. In particular, we tested our hypotheses. Among types of dyad-independent effects, interaction effects control patterns of mixing for categorical nodal attributes. Two ERGM terms were used to test interaction effects [90]. The first effect was uniform homophily, or tendency of nodes with the same attributes to connect (*nodematch*). The term testing this effect adds a statistic for the model for the edges (*i,j*) with equal attribute names. This effect was tested for attributes referring to language, region, and industry. In testing for H1-H3, we would expect to see our model have a positive coefficient indicating a higher than random chance. For H1, a significant and positive coefficient for this term would suggest a higher likelihood of city ties within a region than between regions. Similarly, for H2, a significant and positive coefficient would suggest a higher likelihood of city ties between cities with the same major/official language. For H3, a significant positive coefficient would signal the higher likelihood of city ties between cities specialising in the same industry sector on the international scale. A significant negative coefficient would suggest a higher likelihood for ties to be formed between cities with different regional, linguistic and functional attributes.

Second effect was nodal attribute heterogeneity. The *nodemix* term testing this mixing effect adds a network statistic for each possible pairing (within and between) of attribute values. This effect has been tested for attributes referring to the level of development and a city’s command-and-control role. It tests all potential combinations, through our specific hypotheses are tied to heterophilous relationships. In testing for H4-H5, we would expect to see heterophilous mixing to yield a positive significant coefficient.

We then proceed to fit a model M2 adding to M1 the effects of reciprocity, geometrically weighted edgewise shared partner distribution and the geometrically weighted in-degree and out-degree (which is an approach to model degree distributions and network centralisation [98]). We also added main effects for the largest regional (Europe & Central Asia) and linguistic (English) groups that further improved the model fit.

The model was estimated using Markov Chain Monte Carlo (MCMC) simulation. It considers that the probability of a tie between two cities does not depend only on characteristics of these, but also on network structure. The use of structural attributes aims to capture this effect. A series of network simulations produce parameter estimate and standard error for each variable. The interpretation relies on the logic of a logistic regression, in which the parameter value refers to the log ratio of a given variable in predicting the likelihood of tie formation. Statistical significance is assessed by dividing the parameter estimate by its standard error. Any *p* value smaller than 0.05 suggests a significant relationship, meaning that the hypothesised process exists and predicts network configuration. To assess model fit, we applied the goodness-of-fit test to check whether the models were good representations of the observed network.

The model estimation procedure was implemented in statistical computing environment R. A network file was prepared using *statnet* package in R [99], and five attributes were assigned to each node. We extracted the network backbone using the disparity filter implemented in the *skynet* package [100]. We then used the *ergm* package [101] to specify the composition of models. Our code, along with the input data including edge lists and contingency tables, are available online here: https://osf.io/kcfuh/?view_only=36516ac67ac24bc8b507192523aa7a9c.

## Results

**Fig 1:**
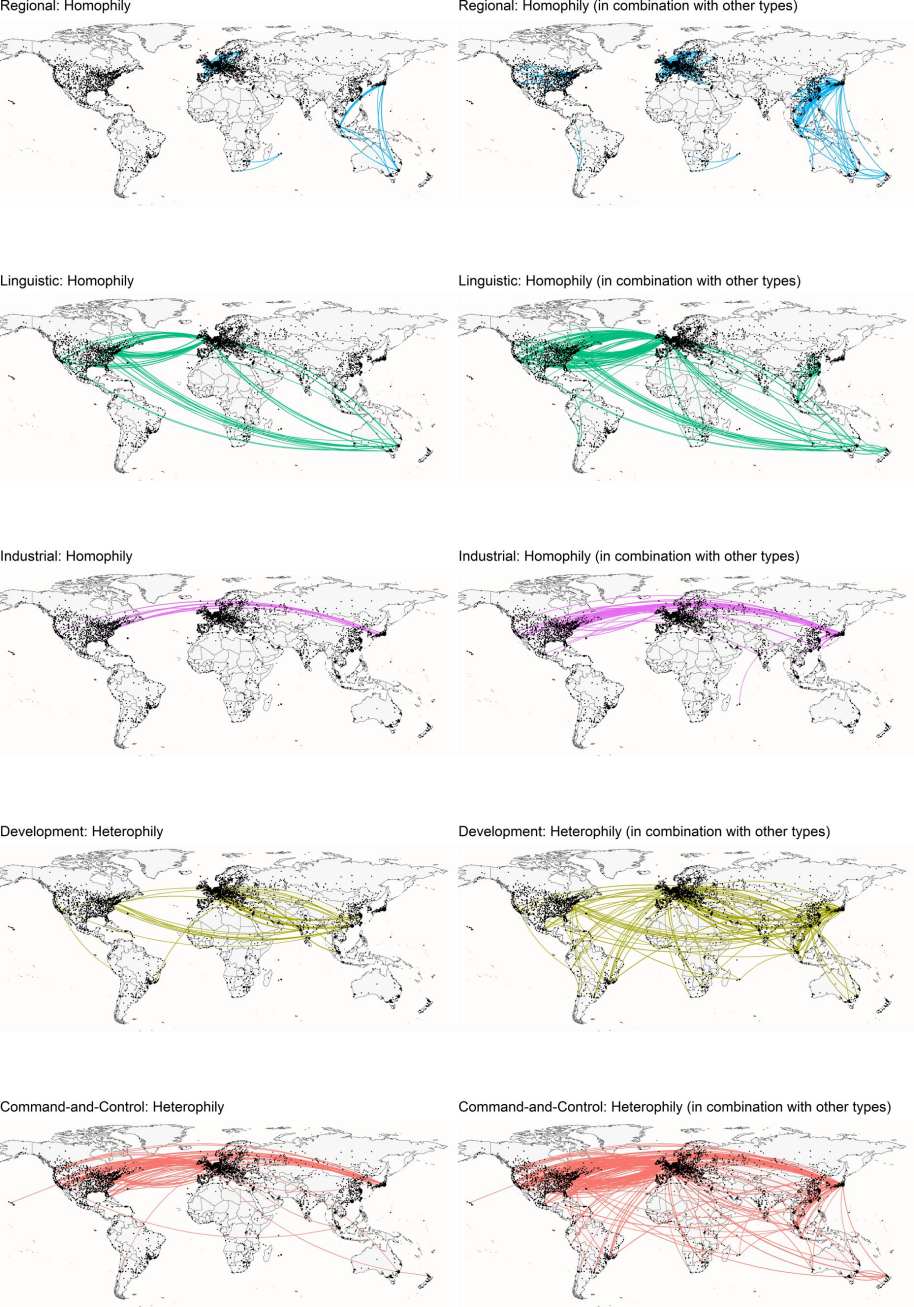
Visual representation of five socio-spatial dimensions explaining dyads in the global city network of firms. Left: ties belonging only to one type. Right: ties belonging to more than one type. Only links with weight more than 30 are displayed. Node size is proportional to its degree.

**Fig 2:**
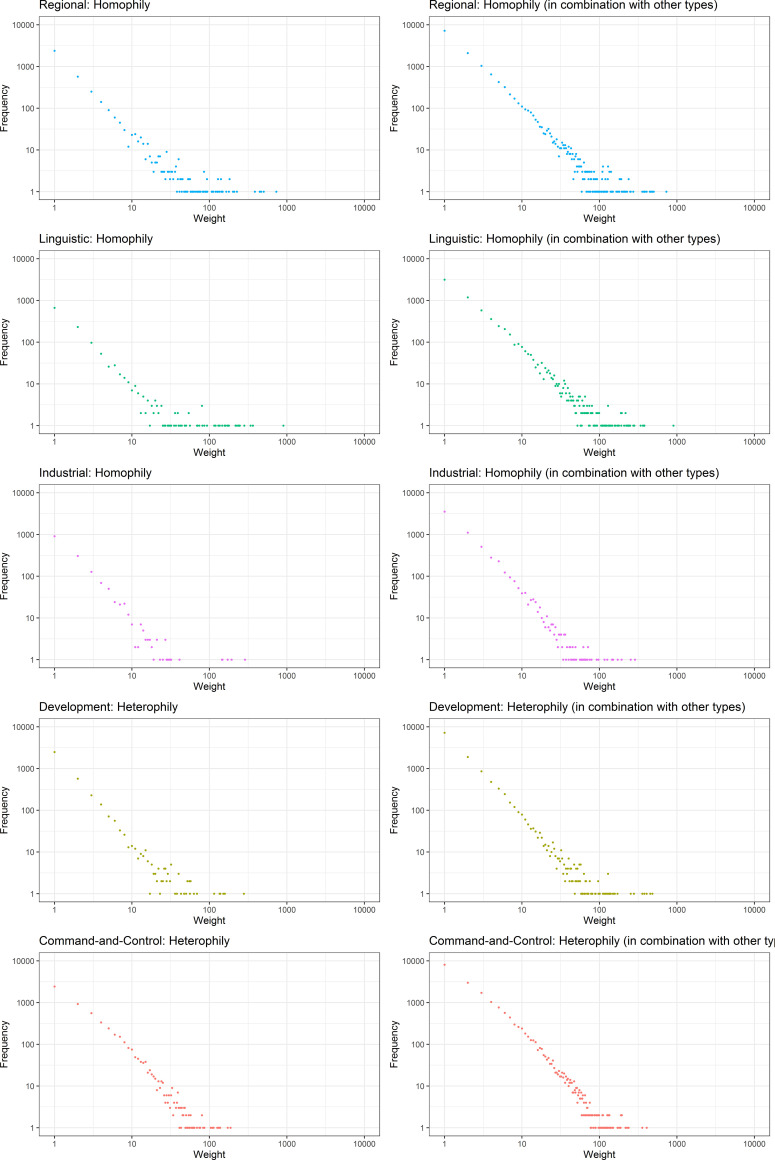
The weight distribution of five types of relations in the global city network of firms. Left: ties belonging only to one type. Right: ties belonging to more than one type.

The global city network of firms that we produced by filtering for international ties only has 38,877 directed weighted edges connecting 3,042 nodes (cities). The different combinations of node-based attributes clearly establish the multirelational character of socio-spatial relations the global city network of firms. These combinations are shown in Fig 1 and the weight distribution is shown in Fig 2. Some ties can be characterised along a single socio-spatial dimension (e.g. ‘regional’ only), whereas a combination of two or more dimensions characterise other ties (e.g., ‘regional and linguistic’, or ‘development, industrial and linguistic’). Indeed, while a tie can be characterised by a single attribute, many of the ties are a result of a combination of dyadic attributes. For example, ties between London and Dublin are simultaneously linguistic and regional, whereas those between London and Rome are only regional.

Fig 2 demonstrates a clear hierarchy in the global city network, with a small number of well-connected cities characterised by ties with high edge weights and vice versa.

**Fig 3:**
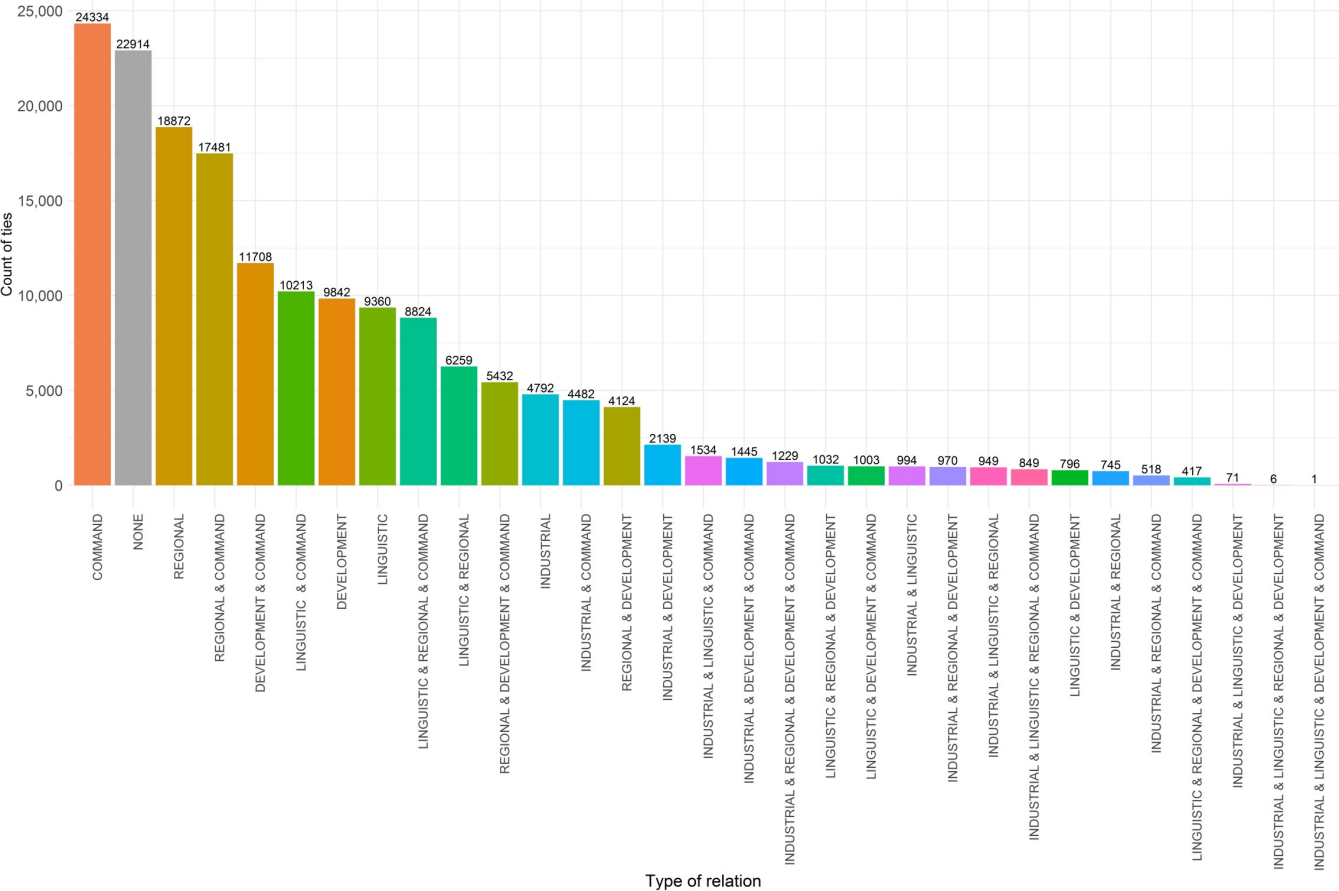
Frequency distribution of different types ties in the global city network of firms.

Within the network, 38.8% of ties were characterised by a single dimension (Fig 2, Left) whereas 48.0% are characterised by two or more (Fig 2, Right). Fig 3 shows the frequency distribution of different ties corresponding to types of relations. The most common type of ties was ‘command-and-control’ ties (n = 24,334). These were followed by ‘regional’ ties (n = 18,872) and ties characterised by a combination of ‘regional and command-and-control’ attributes (n = 17,481). There were 22,914 ties not fitting to any group, indicating other factors might influence their presence. ‘Command-and-control’ ties also existed in combination with ‘development’ and ‘linguistic’ were also prevalent, accounting for n = 11,706 and n = 10,213 ties accordingly. Only 14.0% of dyadic relations can be characterised by a combination of more than two dimensions.

**Fig 4:**
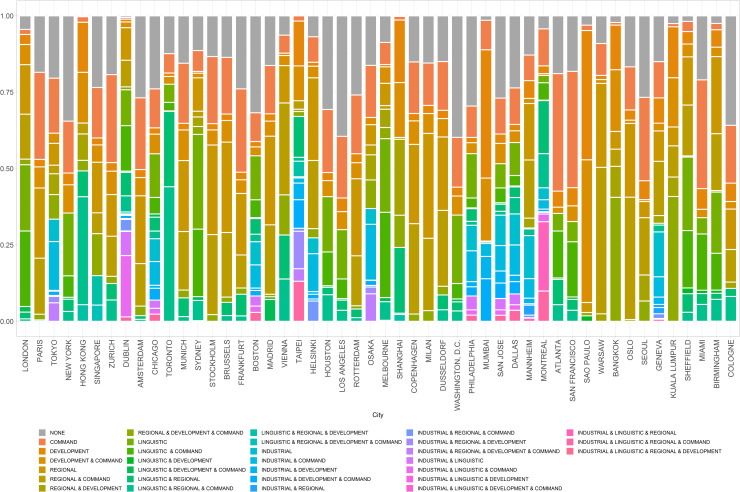
The 50 most connected cities in the global city network of firms by type of relations.

A look at the composition of socio-spatial dimensions for individual city-pairs provides a more complete picture of how and why particular cities are globally connected. Fig 4 demonstrates the breakdown by type of ties for 50 most connected cities in the global city network of firms. This categorisation demonstrates the range of socio-spatial dimensions that characterise global cities whose ties are explained by divergent relations. London is the most internationally connected city in the network with the majority of relations are linked to some combination of linguistic, regional and command-and-control types of relations, driven by the role of London as a world city with extensive connections to cities in Europe and former colonial territories. This is different from Tokyo, whose relations are less linguistic and more industrial and regional, explaining its role of a large world city in Asia and beyond, along with the fact that Japan did not exert cultural influence on overseas territories the way other imperial powers did. Industrial relations are also prevalent in North American cities (Philadelphia and Dallas) as well as in Mumbai, Osaka and Taipei.

**Fig 5:**
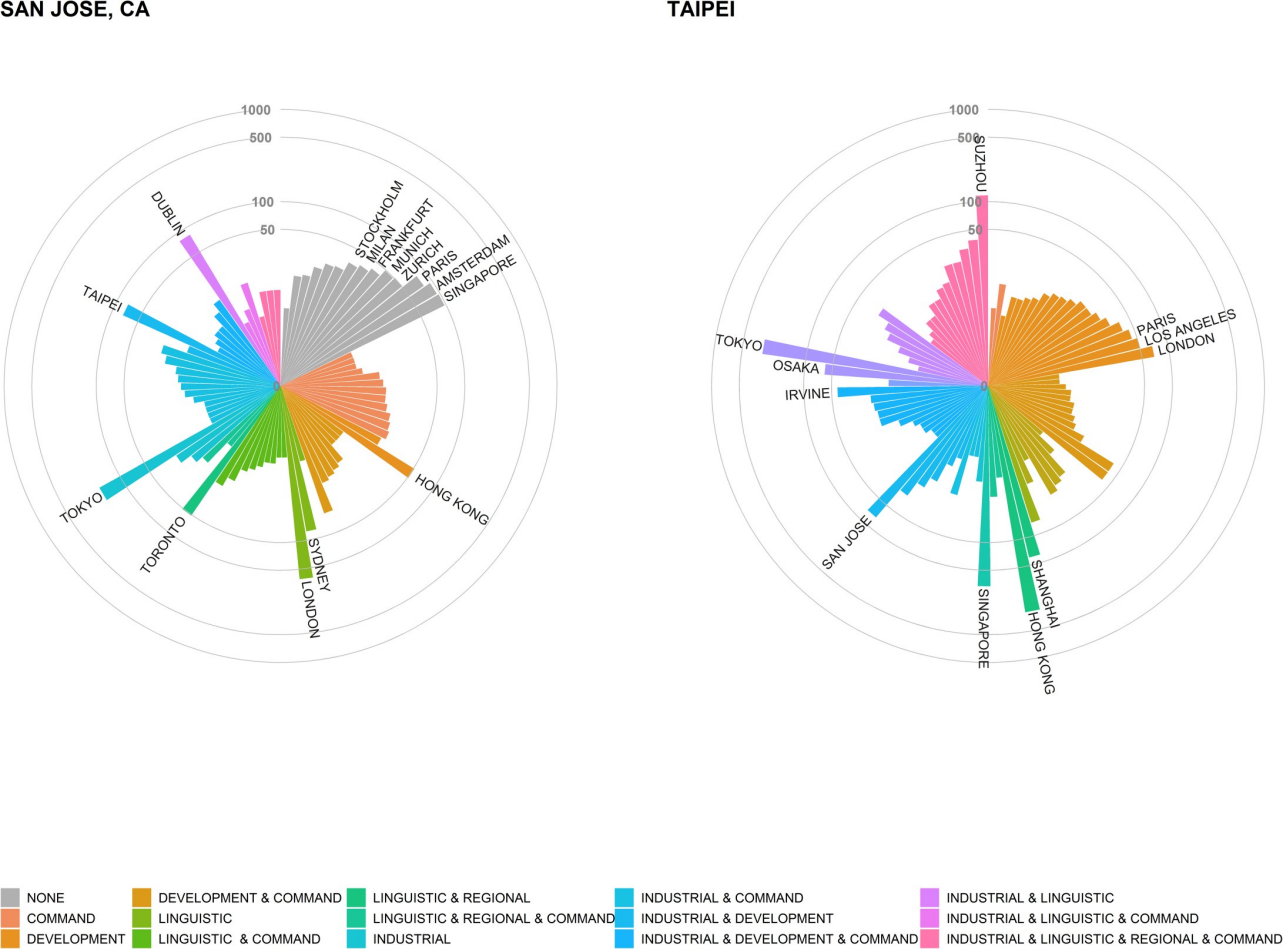
The ego-networks of San Jose, CA and Taipei. Ties with weights more than five are shown. Labels of cities are shown for ties with weights of 20 and higher.

The range of socio-spatial dimensions in Fig 4 provides a visual summary of how diverse cities’ dyadic relations can be. For example, Taipei and San Jose (Silicon Valley) have roughly equivalent connectivity within the global network (ranked as top 20 and 15 most connected cities as shown in Fig 4). Taipei is mostly connected to other cities in East and Southeast Asian nations (e.g. Singapore, China, Thailand, Japan, Malaysia, Indonesia). Strong connectivity to regional cities such as Tokyo, Osaka, and Bangkok is complemented by connectivity to London and Paris and other command-and-control cities. In contrast, San Jose is highly connected through its industrial (Manufacturing) ties, often to other cities in North America and global information and communication technology (ICT) leaders, including Taipei. San Jose is well-connected to cities in the high-tech economies of Switzerland and Israel, and cities with rich technology agglomeration such as Boston, Brussels, and Bangalore. Fig 5 demonstrates how these two cases—San Jose and Taipei—can be compared and contrasted in terms of their dyadic relations (cities) and the socio-spatial variables that explain them (type).

### Exponential random graph models explaining ties in the global city networks of firms

The results of the ERGMs (Table 3) explain the degree to which each socio-spatial dimension explains dyadic relationships. In our null model M0, we used dyad-dependent structural terms *edges* to control for density. In model M1, we added dyad-independent node-based terms: *nodematch* to control for homophily, and *nodemix* to control for heterogeneity (homophily and heterophily). We used *nodematch* to capture the effects of belonging to the same region, language-based homophily and within-industry homophily. We used *nodemix* to capture mixing patterns for income group and command & control position of cities. In model M2, we added *mutual* to control for reciprocity and geometrically weighted out-degree and in-degree distributions (*gwodegree*, *gwidegree*, decay = 0.2) to capture the propensity for centralised network structure, which is a measure of anti-preferential attachment that would be observed in a core-periphery network structure. The term *nodefactor* tests for the main effects for the largest regional (Europe & Central Asia) and linguistic (English) groups. And finally, *gwesp* (decay = 0.1) tests for the general tendency for closure. Each of the ERGMs converged and the goodness-of-fit indicates that our model M2 adequately captures the structure of the reduced global city network of firms (S1 Fig).

**Table 3:** Results of the ERGM to model the probability of inter-city ties as a function of city characteristics.

Model variable (ERGM term)	M0: (edges)	M1: (edges + node-based terms)	M2: (edges + node-based terms + structural terms)	
Predictor	Coefficient	Coefficient	Coefficient	
Density (*edges*)	-4.275*** (0.061)	-6.481*** (0.592)	-6.067*** (0.601)	
HOMOPHILY (*nodematch*)				
Regional	*NA*	-0.097 (0.162)	-0.138 (0.160)	
Linguistic	*NA*	-0.665** (0.256)	-0.189 (0.251)	
Industrial	*NA*	0.420*** (0.126)	0.168^ (0.088)	
HETEROGENEITY (*nodemix*)				
Development (base = Higher–Higher)				
*Lower–Higher*	*NA*	1.182*** (0.180)	0.816*** (0.177)	
*Higher–Lower*	*NA*	0.600** (0.204)	0.364 (0.217)	
*Lower–Lower*		1.556*** (0.194)	0.809*** (0.184)	
Command-and-control (base = High–High)				
*Low–High*	*NA*	1.296* (0.599)	0.960 (0.589)	
*High–Low*	*NA*	0.916 (0.612)	0.735 (0.621)	
*Low–Low*	*NA*	1.440* (0.587)	0.895 (0.567)	
MAIN EFFECT *(nodefactor)*				
*Europe & Central Asia (Region)*	*NA*	*NA*	0.276** (0.092)	
*English (Language)*	*NA*	*NA*	0.090 (0.088)	
Reciprocity (*mutual*)	*NA*	*NA*	2.578*** (0.266)	
Network centralization	*NA*	*NA*		
*Indegree (decay = 0*.*2)*	*NA*	*NA*	-0.002 (0.257)	
*Outdegree (decay = 0*.*2)*	*NA*	*NA*	-1.355*** (0.267)	
Shared partners (*gwesp*, *decay = 0*.*1*)	*NA*	*NA*	1.034***(0.112)	
*AIC*	*2*,*823*	*2*,*697*		*2*,*378*

Note: Standard errors are reported in parenthesis and ρ-values are reported to the right of each coefficient.

^p < 0.1

*p < .05

**p < 0.05

***p < 0.01.

As the ERGM results show, reciprocity is significant and positive, indicating that our observed network has more mutual ties among cities than expected in a randomly generated network. The coefficient for shared partners is positive indicating a propensity for a triadic closure in the network. The indegree anti-preferential attachment coefficient, reflecting a propensity for high-degree cities to receive ties from low-degree cities, is slightly negative but not significant. The outdegree anti-preferential attachment coefficient is negative, reflecting a propensity for high-degree cities to send ties to low-degree cities. This structural term indicates the high degree of network centralization [102].

The ERGM reveals mixed findings for our hypotheses. With regard to homophilous hypotheses, H1 is not supported, in that we cannot observe a uniform effect regarding region, meaning that cities in the same region do not tend to be linked with each other. H2 is no supported either, in that linguistic homophily is not a significant term in the model. This may be because cities in the network core (e.g. London, Amsterdam, Frankfurt, New York, Paris, Tokyo, Beijing) reflect a diversity of languages. In contrast, however, there is support for H3, in that two cities are more likely to be connected if they have similar industry specialisation. This confirms much of the previous literature in global city network research linking places based on economic processes over geographical proximity [21, 70].

Heterophily is found to be an important driver of interurban connectivity within the global city network of firms. With regard to the Development dimension, we support H4 in that cities are more likely to be connected if they belong to countries in a different income group. This effect is positive and significant for Lower-Higher income mixing and positive but not significant for Higher-Lower income mixing. And finally, with regard to the Command-and-Control dimension, the mixing effects Low-High and High-Low are positive but not significant, indicating that the likelihood for cities with different levels of connectivity to connect is not higher than for cities with high levels of connectivity, therefore not supporting H5. The Command-and-Control dimensions is also homophilous (High-High), suggesting that the benefits of connectivity accrue to firms in global cities.

## Discussion and conclusion

As Borgatti and Halgin [19] contend, a large share of the literature on networks focusses on ‘network theory’ rather than the ‘theory of networks’. These are two separate things: one looks at pathways to structures and types of structures—the other looks at the consequences of, and explanations for, those structures. Here, we have sought to augment the latter by providing empirical evidence of how global city networks of firms are explained by a multitude of socio-spatial relations. In doing so, this exploratory analysis provides evidence to suggest that there is a diversity of socio-spatial relations explaining dyadic connectivity between individual cities, and by corollary that network structure is shaped by a diversity of economic processes. The persistent observation that networks exhibit core-periphery structures with a small number of well-connected nodes is supported by our findings.

Global city networks are inherently complex systems produced by diverse actors (e.g. firms, individuals, governments) at multiple scales across both space and time. This study is the first to characterise dyadic relations in the global city network of firms based on the node-based attributes, at least on such a scale. Beyond contributing to the global city networks literature, we address the common critique that network approaches are methods-rich, but contain very little social theory, or rather that scholars tend to focus on its refinement rather than its application. In this instance, we apply innovations in network science toward a better understanding of how cities are embedded within socio-spatial processes that underlie economic relations, and ultimately, the global economy. As Ward et al. [103] note “network analysis is more than a tweak to the status quo ante; rather, it offers a means of addressing one of the holy grails of the social sciences: effectively analysing the interdependence and flows of influence among individuals, groups, and institutions.” (p. 245).

This study confirms that dyadic relations within global city networks of firms are explained by more than one nodal attribute. The examples of Taipei and San Jose (Silicon Valley) demonstrate how two cities can be roughly equivalent in their aggregate connectivity, yet dramatically different in the makeup of their network embeddedness. Whereas Taipei plays a strong regional role, San Jose’s network relations are explained more by industry-specific ties derived from its leadership in global ICT.

The results of the ERGM reinforce several of our hypotheses, namely that similarities in industry composition (homophily) play a strong role in determining interurban connectivity, and that cities in lower-income countries, and cities with subsidiary locations are more dependent on network ties than vice-versa. That is to say, we find that a core-periphery structure really determines the network’s behaviour.

Although these five socio-spatial dimensions in this study are not exhaustive, their application serves as foundational to this exploratory study. Future research may look to refine or augment these dimensions, or to investigate each in further detail. One possible avenue of exploration is to extent this approach to valued networks and valued ERGMs that might require more complex specifications of the reference terms. Additionally, though the hierarchies inherent to global city networks of firms are well-documented, a focus on the ‘long tail’ of the urban distribution may reveal how and why smaller cities, poorly connected cities, and/or cities in less-developed regions are able to leverage tie formation through local firms to participate in global economic networks.

## Supporting information

S1 Fig ERGM goodness-of-fit (GOF) diagnostics. (PDF)
