# Supplementary material for: Socio-spatial relations observed in the global city network of firms
Source: PLoS One. 2021 Aug 17;16(8):e0255461. doi: 10.1371/journal.pone.0255461 (PMC8370647; doi:10.1371/journal.pone.0255461)
Supplement: S1 Fig — (PDF) [file pone.0255461.s001.pdf]

S1: ERGM goodness-of-fit (GOF) Diagnostics

The following plots demonstrate the results of the GOF tests for our ERGM M2. The plots show that the ERGM converged and was not degenerate, reflecting the ability of the model to adequately capture the observed structure of our empirical global city network of firms (reduced).

Figure 1: Goodness-of-fit plots (in degree, out degree, edge-wise shared partners and minimum geodesic distance) for the ERGM M2 model.

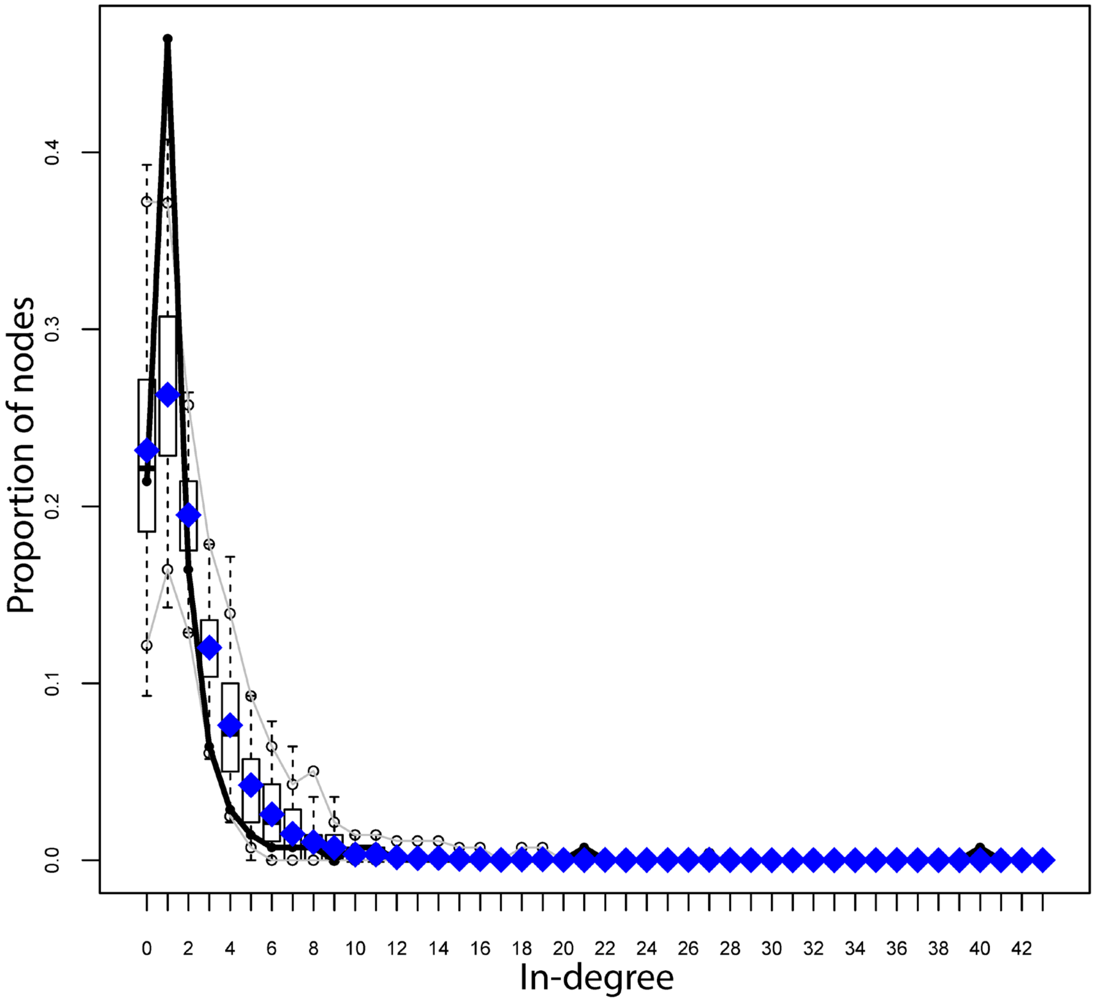

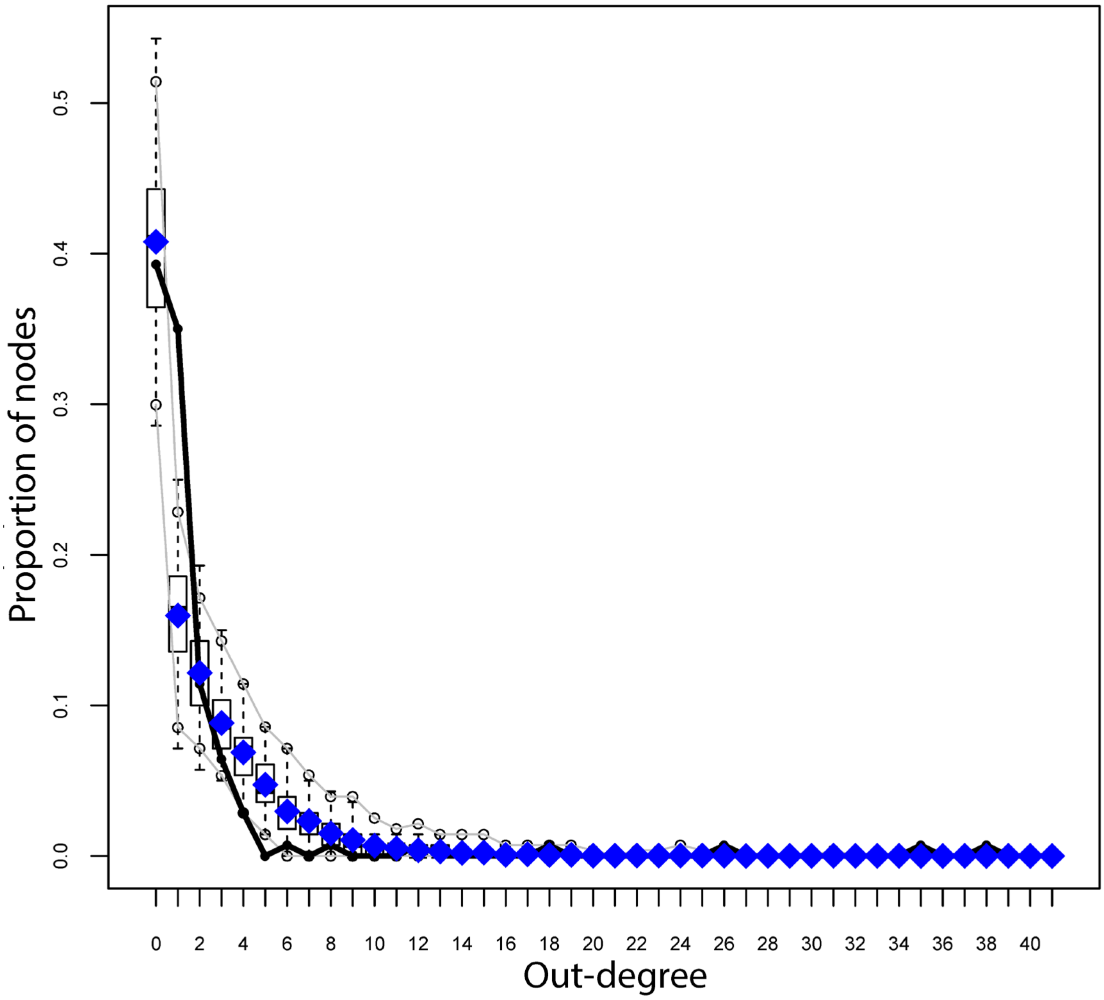

Proportion of nodes
0.0
0.1
0.2
0.3
0.4
0.5
0 2 4 6 8 10 12 14 16 18 20 22 24 26 28 30 32 34 36 38 40
Out-degree

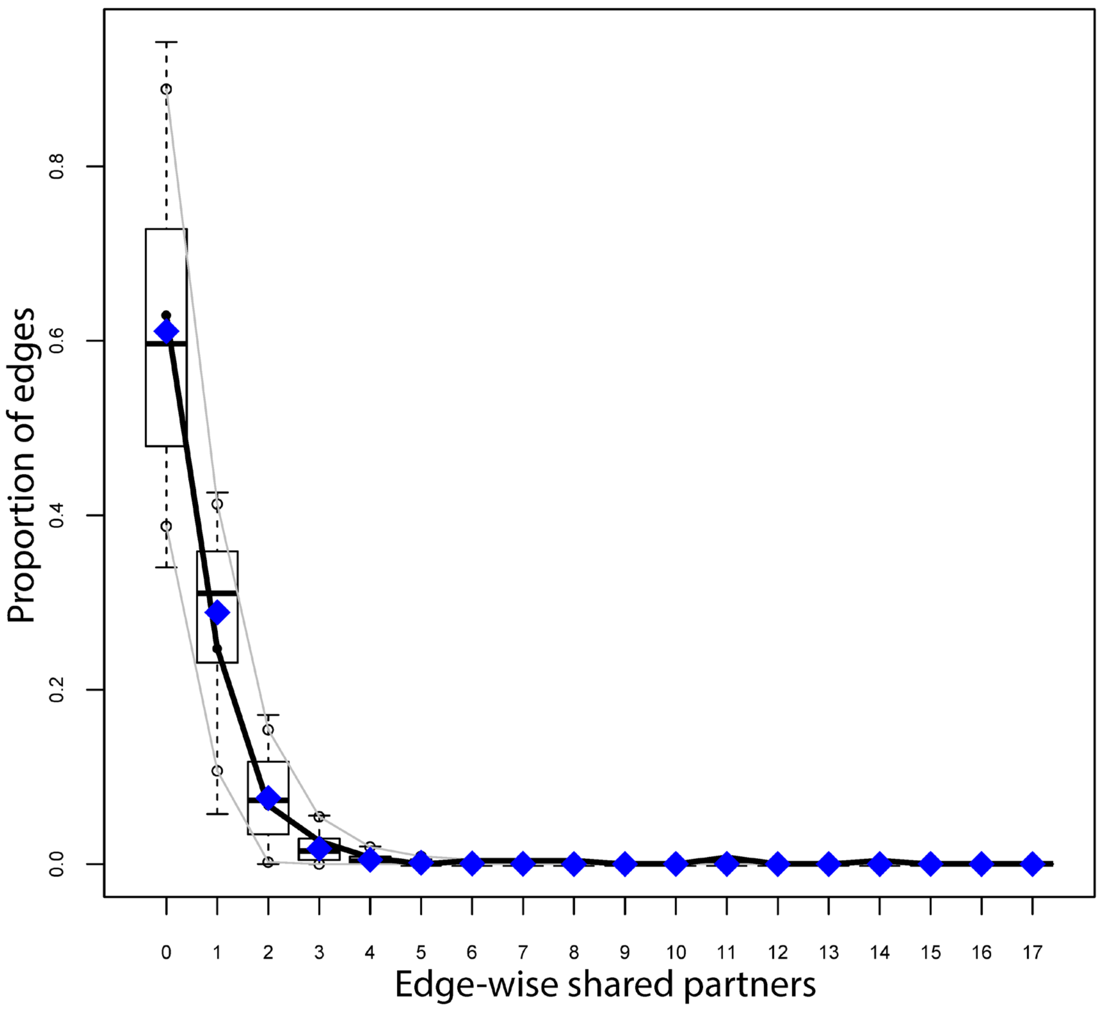

Proportion of edges
0.0
0.2
0.4
0.6
0.8
0
1
2
3
4
5
6
7
8
9
10
11
12
13
14
15
16
17
Edge-wise shared partners

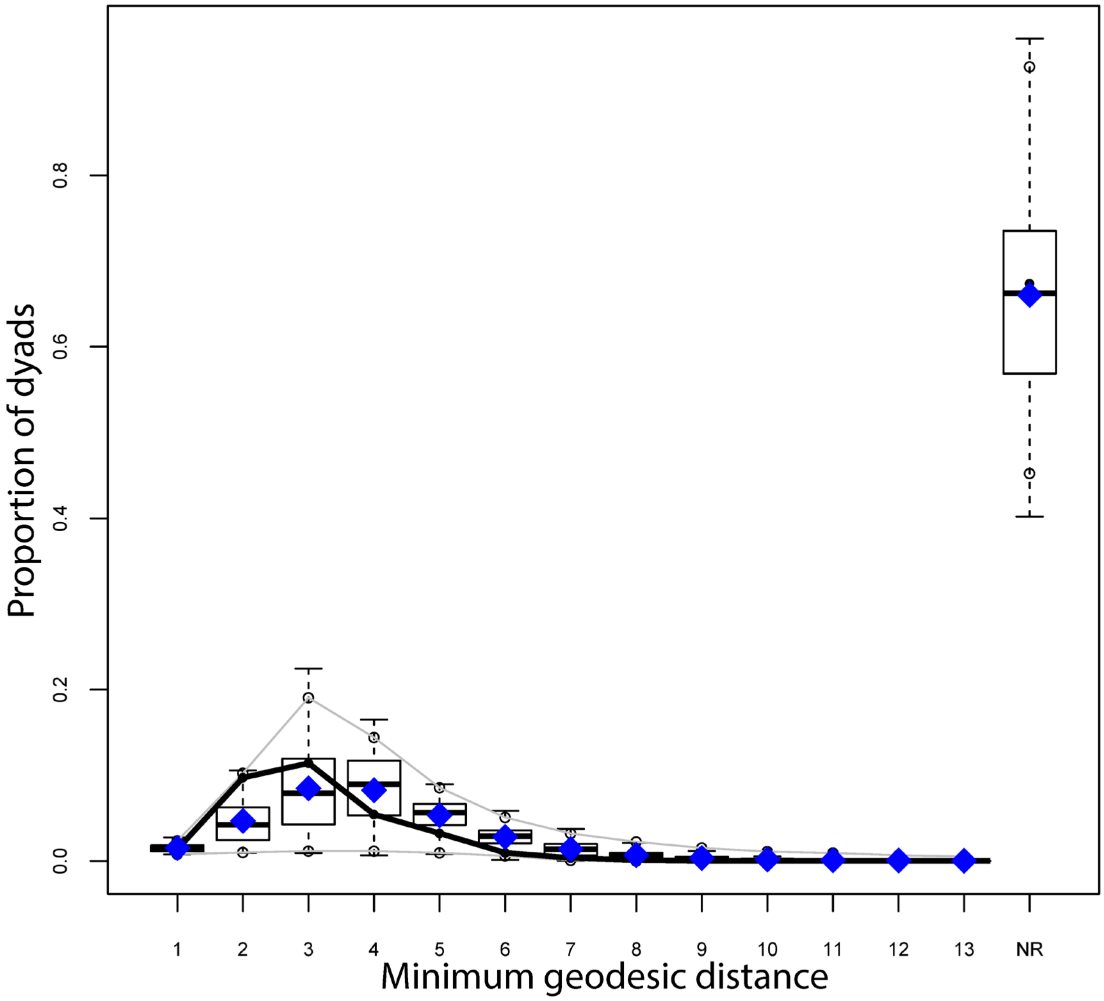

Proportion of dyads
0.0
0.2
0.4
0.6
0.8
1
2
3
4
5
6
7
8
9
10
11
12
13
NR
Minimum geodesic distance
